# Pandemic Babies: Developmental Outcomes in Preschool-Aged Children Born During the COVID-19 Era

**DOI:** 10.3390/bs16020309

**Published:** 2026-02-23

**Authors:** Sally Sade, Claudia L. R. Gonzalez, Robbin L. Gibb

**Affiliations:** Canadian Centre for Behavioural Neuroscience, University of Lethbridge, Lethbridge, AB T1K 3M4, Canada; claudia.gonzalez@uleth.ca (C.L.R.G.); gibb@uleth.ca (R.L.G.)

**Keywords:** COVID-19, pre-pandemic, pandemic, executive function, developmental domains, preschool

## Abstract

Early life experiences and the process of exploration play a vital role in shaping brain development and lifelong learning. In March 2020, population-wide restrictions were imposed due to the COVID-19 pandemic. It remains to be determined whether having been raised under the global stress and restrictions of COVID-19 has influenced children’s development as they enter formal schooling. The aim of this study was to examine the extent to which having more than 50% of one’s first year of life and/or prenatal period in the COVID-19 era influences the developmental trajectory in preschool. The study compared 3- to 5-year-old children born before the pandemic (*n* = 63) with those who were five months or younger at its onset (*n* = 40). Variables assessed included executive function skills, vocabulary, and common developmental domains. Using the BRIEF-P as a standardized measure of executive function, the results demonstrate that the pandemic-born cohort exhibit greater impairments than those born before the pandemic. There was also a significant increase in reports of speech and language therapy enrollment; frequent ear infections; diagnoses of hearing, speech, or language impairments; and delays in reaching developmental milestones. The pandemic-born cohort additionally reported delays in fine motor skills compared to the pre-pandemic cohort. The present study underscores the urgent need for additional resources to better support children in this cohort as they begin formal schooling.

## 1. Introduction

Early childhood, particularly from birth to around two years of age, is often regarded as a “window of opportunity,” during which experiences shape lifelong health, behavior, and learning trajectories (UNICEF, 2017). This is in part due to the rapid and steep changes in structural brain development during the prenatal and early postnatal period (Gilmore et al., 2018). While prenatal development is mainly driven by genetics, gene expression is influenced by the maternal environment, including factors such as inadequate nutrition, toxin exposure, and elevated cortisol (Tierney & Nelson, 2009). Postnatally, brain development becomes increasingly shaped by the dynamic interplay between genetic predisposition and environmental experience. Longitudinal neuroimaging studies have begun to delineate these early maturational processes (Almli et al., 2007). At term birth, the brain is remarkably organized, with a developed white-matter connectome and distinct cortical structures in place (Cao et al., 2016; Gilmore et al., 2018). Over the first year, cortical gray matter and white matter microstructure develop, alongside the emergence of higher-order functional networks at rest (Gao et al., 2013). By age two, the brain’s core structural and functional architecture is in place, laying the foundation for long-term cognitive and behavioral outcomes (Gilmore et al., 2018).

The developing brain is susceptible to and in turn shaped by a myriad of experiential factors, ranging from the physical environment, whether natural or human-made, to the complex web of social interactions with caregivers, family, and peers. A growing body of literature supports the notion that both the timing and type of early experiences can exert lasting effects on cognitive, behavioral, and emotional outcomes (Kundakovic & Champagne, 2015; Rutter, 1980). For instance, exposure to adverse childhood experiences (ACEs) prior to the preschool years elevates the risk of poor school readiness (Jackson et al., 2021; Shen et al., 2025). The effects of these experiences appear to be intergenerational. Higher levels of parental ACEs have been associated with poorer preschool readiness (Shen et al., 2025) and academic performance (Doi et al., 2020). However, parental resilience, defined as a parent’s capability to cope with stressors affecting everyday life and/or stressors related to their child, can influence parenting behaviors and child outcomes (Kiplinger & Browne, 2014; Verhagen et al., 2025). In addition, children’s psychological resilience has been shown to play a mitigating role in the association between parental ACEs and both school engagement (Bethell et al., 2014) and preschool readiness (Shen et al., 2025). Natural disasters such as the 1998 Quebec ice storm have offered unique opportunities to study the effects of prenatal maternal stress on child outcomes as a “natural experiment.” Laplante et al. (2004, 2007) demonstrated that high levels of maternal stress during the first or second trimester were linked to poorer cognitive and language outcomes in children at two years of age. Contrary to the notion of developmental “catch-up”, these effects were not transient; follow-up studies at 5 ½, 8 ½, and 11 ½ years revealed that these delays persisted, notably in males (King & Laplante, 2015; Laplante et al., 2008). Structural brain scans at 11 ½ years showed that males exposed to high levels of objective prenatal maternal stress displayed smaller normalized right hippocampal volumes (Cao-Lei et al., 2021). There was also evidence that postpartum exposure to maternal stress, likely through breastmilk, was associated with smaller left hippocampal volume in both sexes (King & Laplante, 2015). Severe early-life deprivation tells a similar story. The Bucharest Early Intervention Project highlighted the consequences of neglect and lack of high-quality experiences in the early years. Children who spent their first two years or more in institutional care exhibited long-lasting deficits across multiple domains of development (Johnson, 2000). Whereas children who were adopted at 6 months or earlier displayed better outcomes (Rutter, 1998). Later studies (Berens & Nelson, 2015; Nelson et al., 2007; Smyke et al., 2012) extended the sensitive period to 24 months, rather than 6 months, noting that periods of deprivation longer than 24 months result in significant developmental abnormalities.

Similarly, albeit at a global scale, the COVID-19 pandemic presented significant challenges for expecting parents and newborns due to an unprecedented shift in health and environmental conditions. Although the World Health Organization (WHO) declared COVID-19 a pandemic on 11 March 2020, its course unfolded in multiple waves, varying in intensity and public response (Dam et al., 2023). Awareness of the virus began in December 2019, and it was declared a public health emergency of international concern on 30 January 2020 (WHO, 2020). In Canada, January 2021 saw the highest average number of deaths, while January 2022 marked the month with the highest average number of reported cases (Public Health Agency of Canada, 2022). As in many other provinces, families in Alberta experienced intermittent lockdowns and changing public health restrictions until March 2022, when restrictions were lifted. Across this two-year period, challenges such as limited access to healthcare and education, reduced social interactions, and exposure to prolonged stress were exacerbated. Expecting parents during the pandemic reported heightened concerns related to birth support, prenatal care access, infection risk, and not being able to introduce their baby to loved ones (Bogler et al., 2021). Parents of infants born during the pandemic described their experience as lonely and isolating (Sledge et al., 2022), and rates of maternal psychological distress were reported to be 3–4 times higher than pre-pandemic baselines (Lebel et al., 2020). Consistent with these reports, pandemic-related stressors were associated with a decline in maternal health (Jaffer et al., 2024). Furthermore, these elevations in distress occurred irrespective of COVID-19 symptomatology and were instead attributed to reduced social support (Provenzi et al., 2023). Understanding how exposure to these environmental shifts has influenced the developmental processes of a generation of infants is of considerable policy interest.

Emerging evidence suggests that developmental differences in children exposed to the pandemic become more apparent later in infancy, particularly around 12 months of age, with notable delays in communication and fine motor skills. Findings at six months are mixed: while Shuffrey et al. (2022) reported lower gross motor, fine motor, and personal-social scores using the Ages and Stages Questionnaire (ASQ-3), other studies found no significant differences at this age (Huang et al., 2021; Imboden et al., 2022). By 12 months, however, several studies have documented developmental concerns in this population. Huang et al. (2021) observed increased risk of delay in communication and fine motor skills, with communication delays limited to first-born children. Giesbrecht et al. (2023) reported lower mean scores and increased odds of screening positive for delay in communication, gross motor, and personal-social domains. Byrne et al. (2023a) also documented reduced social communication scores in this population. A follow-up study at 24 months identified lower scores on communication and gross motor skills (Byrne et al., 2023b); however, no significant differences were observed in other developmental domains. Timing of exposure also seems critical. Sato et al. (2023) found that children exposed to the pandemic at age three years showed significant developmental delays by age five, while those exposed at age one did not differ from their peers at age three.

As children born during the pandemic go through preschool, concerns are emerging regarding their school readiness, particularly in domains that rely on executive function (EF). EF is a construct that encompasses multiple interrelated higher-order cognitive processes required for self-regulation, flexible problem-solving, and goal-directed behavior (Diamond, 2013). According to the Unity and Diversity model (Miyake et al., 2000), EF consists of three discrete but interrelated “core” mental skills: (1) working memory—the ability to retain and manipulate information to guide ongoing behavior; (2) inhibitory control—the ability to resist prepotent responses or distractions; and (3) shifting—the ability to swiftly adapt to changing task demands. This framework suggests that higher-order EFs such as planning, problem-solving, and organizational skills are built upon these core executive processes. As discussed in Stuss et al. (2002), brain injury research has linked the development of EF to the prefrontal cortex (PFC), one of the last brain regions to mature. However, although the PFC serves as a major neural substrate of EF performance, its function depends on extensive bidirectional connectivity with other brain regions (Buchsbaum et al., 2005; Heyder et al., 2004; Rottschy et al., 2012). Interestingly, EF is among the most frequently reported cognitive domains affected by early environmental adversity (Blair & Raver, 2016). Furthermore, research indicates a relationship between childhood EF and later outcomes in health, wealth, and crime, with lower EF associated with a higher prevalence of negative outcomes (Moffitt et al., 2011). Given EF’s known vulnerability to early-life stress and its foundational role in cognitive development (Center on the Developing Child at Harvard University, 2011), it represents a critical functional domain for assessing potential pandemic-related developmental impacts.

Many studies have focused on the impact of EF skills in the preschool-age population exposed to COVID-19, largely due to the loss of access to early childhood education programs, support services, and increased social isolation. These studies have reported reduced school readiness (González et al., 2022), increased dysregulation during the lockdowns (Di Giorgio et al., 2021; Hanno et al., 2022), and a reduction in social cognitive skills (Scott et al., 2024). To date, no studies have reported EF outcomes in preschool-aged children born during the COVID-19 pandemic. Furthermore, prior research has several limitations, including reliance on parent-report measures and lacking a contemporaneous pre-pandemic comparison group, often using historical cohorts instead (see review by Alcon et al., 2024). The current study addresses these gaps by comparing children born during the pandemic to a pre-pandemic cohort drawn from the same ongoing study. Specifically, we leverage data from the Building Brains Together (BBT) program, a community-based initiative designed to support EF development through adult-directed play. Moreover, whereas many studies were published while the pandemic was ongoing, the present study assesses the effect post-pandemic. Based on prior research, we aimed to assess how an early pandemic exposure window may impact developmental outcomes at preschool age. Our predictions were as follows. First, given the importance of early environmental experiences, we predicted that the pandemic-born cohort would demonstrate poorer developmental milestone achievement, based on the participant information sheet, relative to their pre-pandemic peers. Second, consistent with these environmental disruptions, we predicted that the pandemic-born cohort would report poorer outcomes on the BRIEF-P, ASQ-3 and ASQ:SE-2, which are standardized measures of EF and developmental functioning. Third, we expected that these effects would be moderated by perceived stress and parental resilience, as measured by the ACEs survey and the PAPF, respectively.

## 2. Materials and Methods

### 2.1. Participants

This cross-sectional study is part of a larger BBT project; an ongoing study assessing how adult-directed play can improve EF skills in children. Between April 2022 and June 2025, participants were recruited from a total of 14 daycares and preschools throughout Southern Alberta. Advertisements and posters were shared with eligible participants via mass emailing, social media, and word of mouth. Eligibility criteria included being between 3 years and 5 years and 11 months of age and having normal or corrected-to-normal vision and hearing. Once consent was obtained from the caregiver, children were tested at their respective daycare or preschool under the supervision of site staff. This study (Pro00120933) was reviewed and approved by the research ethics board at the University of Alberta and followed the Strengthening the Reporting of Observational Studies in Epidemiology (STROBE) reporting guideline (Vandenbroucke et al., 2007).

**Figure 1:**
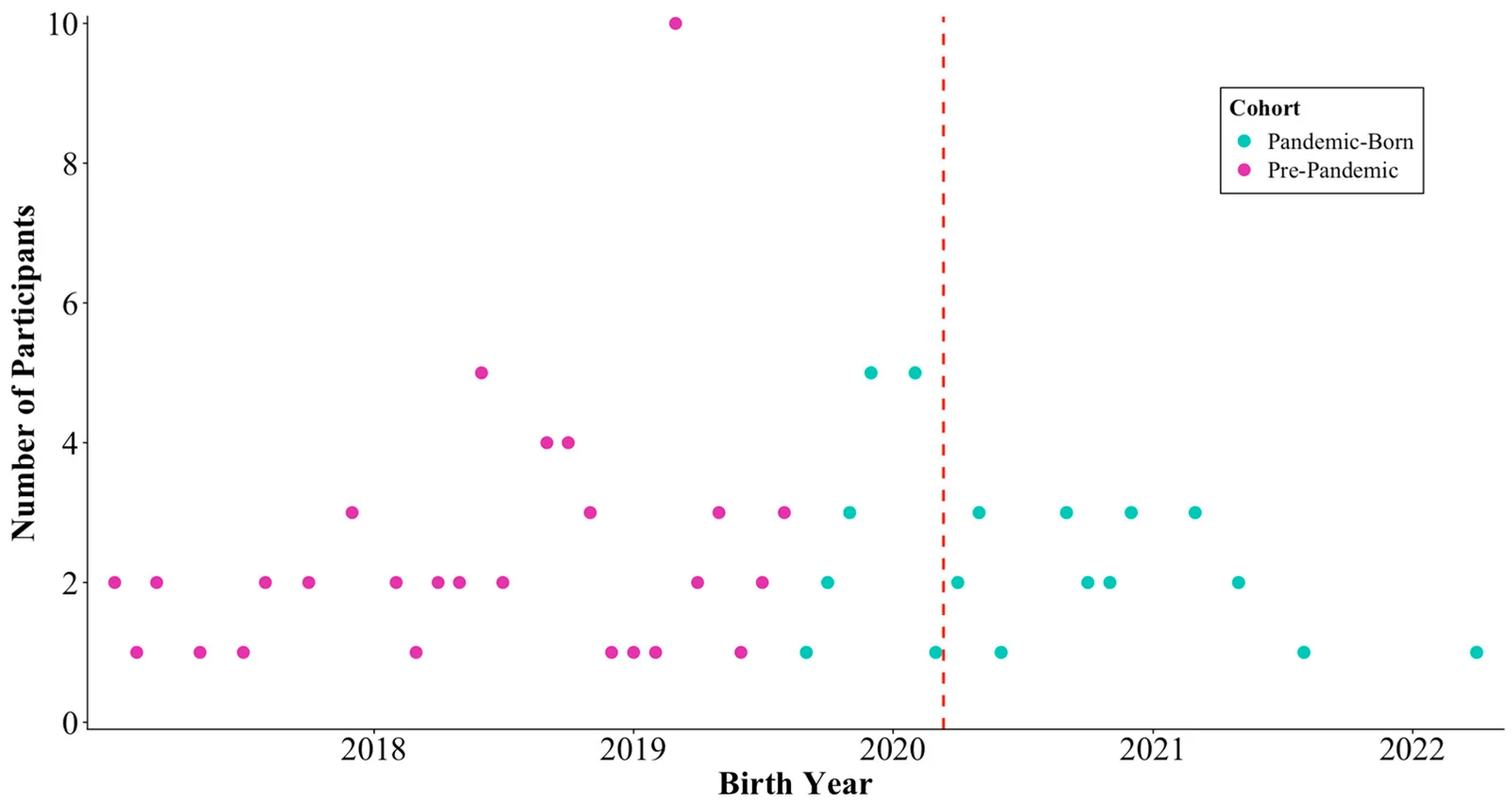
Birth year distribution of the study population relative to COVID-19 pandemic start. *N* = 103 (pre-pandemic = 63; pandemic-born = 40). The dashed line represents the date, 11 March 2020, at which COVID-19 was assessed as a pandemic.

This study included 103 preschool children (63 pre-pandemic, 40 pandemic-born). The demographic characteristics of the participants are shown in Table 1. The sample was predominantly Caucasian (72%). According to the 2021 Census of Canada, 73.9% of Albertans identify as white/Caucasian, indicating that the sample demographic makeup was consistent with provincial demographics. Educational attainment in the sample was higher than provincial averages, with 60% of caregivers holding a bachelor’s degree or higher, compared to 31.1% of Albertans (Statistics Canada, 2021). The pandemic-born group was defined as children who lived the first 50% of their life during COVID-19; specifically, those who were either in utero or up to five months old at the onset of the pandemic (March 2020). The pre-pandemic group was defined as children who were six months or older at the start of the pandemic. The cutoff was established to consider and address important developmental milestones that occur during the first year of life. The birth distribution relative to pandemic exposure of the sample is shown in Figure 1.

### 2.2. Procedure

Consent was obtained from participating center directors, early childhood educators, and caregivers. Questionnaire packages were distributed to caregivers by site staff, to be completed at their own convenience and returned within two weeks. The questionnaires included: (a) a parent information sheet; (b) Behavior Rating Inventory of Executive Function–Preschool Version^TM^ (BRIEF-P^TM^; Gioia et al., 2003); (c) the Parents’ Assessment of Protective Factors (PAPF; Kiplinger & Browne, 2014); (d) the Ages and Stages Questionnaire-Third Edition (ASQ-3; Squires & Bricker, 2009); (e) the Ages and Stages Questionnaire: Social Emotional-Second Edition (ASQ:SE-2; Squires et al., 2015); (f) the Adverse Childhood Experiences Questionnaire (ACE-Q; Felitti et al., 1998) self-report and child-report. Given the sensitive nature of some questionnaires, caregivers were informed that they could skip any items they found uncomfortable without penalty or removal from the BBT program. As an incentive for participation, families received a Building Brains Together Game Bag (Coelho et al., 2020), which includes a set of games designed to support the development of EF skills in preschool-aged children.

Language assessments were administered individually in a quiet location within each child’s daycare or preschool. Assent was obtained from each child prior to assessment. Upon completion, children received a sticker as a reward. Assessments were conducted in a single session lasting approximately 30–45 min. However, if the child expressed fatigue or requested to stop, the session was immediately halted and resumed on a later day. All assessments were administered by trained graduate and undergraduate research assistants who demonstrated acceptable interrater reliability prior to data collection.

### 2.3. Measures

#### 2.3.1. Participant Information Sheet

The participant information sheet was developed by the Building Brains Together research group. It collected general demographic information, screen time reports, and motor, executive function and language characteristics. In addition, we asked caregivers to report on their pregnancy experience before, during, and after pregnancy, as well as their child’s common developmental milestone screening as assessed by the Health Unit at vaccination appointments. These items were limited to general developmental milestones and were not standardized assessments. The participant information sheet consisted of a mixed-response format in which participants answered closed-ended yes/no questions and open-ended prompts that allowed for further elaboration. This information was collected for descriptive and eligibility purposes (questionnaire is provided in Supplementary Material S1).

#### 2.3.2. Behavior Rating Inventory of Executive Function—Preschool Version

Caregivers reported their children’s executive function strengths and weaknesses using the BRIEF-P (Gioia et al., 2003). This questionnaire consists of 63 items rated on a three-point scale (never, sometimes, often) that assess five executive function domains: Inhibit, Shift, Emotional Control, Working Memory, and Plan/Organize. These clinical scales form three broader indices: Inhibitory Self-Control (ISCI), Flexibility (FI), and Emergent Metacognition (EMI). Together, the five domains yield an overall composite score, the Global Executive Composite (GEC). The current study used *T* scores (normative *M* = 50, *SD* = 10), with higher scores indicating poorer EF (Gioia et al., 2003). Internal consistency on subscales is: Inhibit α = 0.90, Shift α = 0.85, Emotional Control α = 0.86, Working Memory α = 0.88, Plan/Organize α = 0.80, and Global Executive Composite α = 0.95. The BRIEF-P contains two additional scales to measure validity, the inconsistency and negativity scale. The inconsistency measures the extent to which caregivers rate similar items in an inconsistent manner. The negativity scale measures the degree to which parents rated items as *Often* across 10 items that compose the negativity scale. Parent ratings with a Negativity raw score of 4 or more are suggested to be reviewed carefully and may indicate an excessively negative perception on the part of the rater.

#### 2.3.3. Parents’ Assessment of Protective Factors

The PAPF was used to assess four protective factors: (a) parental resilience, (b) social connections, (c) concrete support systems in times of need, and (d) social and emotional competence of children (Kiplinger & Browne, 2014). Each subscale consists of nine items rated by caregivers on a 5-point Likert scale ranging from 0 (This is not at all like me or what I believe) to 4 (This is very much like me or what I believe). Subscale scores were computed by summing the item responses, and an average score was calculated for each subscale. A Protective Factors Index (PFI) was calculated across all four subscales. Average scores for the subscales and the index can be interpreted as Low (0–1.99), Moderate (2.00–2.99), High (3.00–3.99), and Maximum (4.00), with higher scores indicating stronger caregiver beliefs, feelings, and behaviors regarding that protective factor. Internal consistency (α) for the protective factor subscales ranged from 0.88 to 0.93, with the overall PFI at α = 0.95.

#### 2.3.4. Ages and Stages Questionnaire—Third Edition

The ASQ-3 is a standardized age-specific development screening tool used to detect or predict delays across five domains: (a) communication, (b) gross motor, (c) fine motor, (d) problem solving and (e) personal-social. Each subscale consists of six items (30 items total). Responses to the six items within each subscale are summed to generate a subscale score. Responses are scored as follows: yes = 10 points, sometimes = 5 points, and not yet = 0 points. Higher scores reflect better developmental progress. Internal consistency (α) for the ASQ-3 across the 36- to-60-month intervals ranged from 0.61 to 0.83.

#### 2.3.5. Ages and Stages Questionnaire: Social Emotional—Second Edition

The ASQ:SE-2 is an extension of the ASQ, focused on providing a more thorough understanding of the children’s social-emotional development (Squires et al., 2015). The ASQ:SE-2 is also age-specific and consists of 36 items. Caregivers indicate whether each statement best describes their children’s behavior often/always, sometimes, or rarely/never. Each response is coded with a letter (Z, V, or X), with the letter’s numerical value dependent on the specific item (e.g., Z may correspond to rarely/never or often/always). Responses are scored as follows: Z = 0 points, V = 5 points, and X = 10 points. Caregivers also indicate whether each item is an area of concern; if so, an additional five points are added to the score. Lower total scores indicate stronger social-emotional development. Internal consistency (α) for the ASQ:SE-2 ranged from 0.71 to 0.90, with an overall alpha of 0.84, across age intervals.

#### 2.3.6. Adverse Childhood Experiences Questionnaire

The ACEs questionnaire is a brief rating scale adapted from Felitti et al. (1998) used in this study to capture early-life adversity. The questionnaire consists of 10 items covering categories such as abuse, neglect, and household dysfunction. Caregivers were asked to complete a self-report on the adverse childhood experiences they encountered between 0 and 18 years of age. Additionally, they completed the same report on behalf of their participating child (ren). The total ACE score represents the number of items the caregiver or their child experienced (scored as +1), with higher scores indicating greater cumulative risk.

#### 2.3.7. Vocabulary Assessment

Examiners administered the Peabody Picture Vocabulary Test—Fifth Edition (PPVT-V; Dunn, 2019) a standardized tool used to assess receptive vocabulary skills. Children were instructed to select a single picture out of the four available options that best described the object or action named by the examiner. Standard scores were used for statistical analyses, with higher scores denoting better language competence. Internal consistency (*r_sb_*) ranged from 0.94 to 0.98 (*M* = 0.97).

### 2.4. Data Analysis

Statistical analyses and visualizations were conducted using RStudio (Posit Team, 2025, version 2025.05.1+513). Descriptive statistics (mean, median, interquartile range, and standard deviation) were used to describe cohort characteristics across various covariates (age at time of testing, sex, household composition, etc.). The scores across all measures were analyzed between the pandemic-born and pre-pandemic cohorts. Prior to analysis, the parent-reported questionnaires and language assessment were checked to determine if they met the assumptions of parametric statistics. Normality and homogeneity of variance were evaluated using the Shapiro–Wilk test and Levene’s test, respectively. Firstly, to determine whether item responses on the participant information sheet differed between the two cohorts, we conducted a chi-square test of independence. Measures that met the assumptions of a parametric test were analyzed using a Welch’s two-sample *t*-test and ANCOVA, whereas the Mann—Whitney U test and Spearman’s correlation, non-parametric tests, were employed for tasks where assumptions could not be confirmed. A sensitivity power analysis conducted in G*Power 3.1 indicated that, with group sizes of *n* = 55 (pre-pandemic) and *n* = 38 (pandemic-born), α = 0.05 (two-tailed), and power = 0.80, the study was powered to detect a minimum effect size of Cohen’s *d* = 0.61 for between-group comparisons. Due to varied sample size across analyses, this sensitivity estimate is based on the smallest analytic sample, providing a conservative estimate of detectable effects.

## 3. Results

### 3.1. Sociodemographic and Early Clinical Factors Across Cohort

At the first stage of analysis, we assessed whether group differences would be observed across caregiver-reported background factors. These included information gathered from the participant information sheet, PAPF, and ACEs. A total of 103 caregivers received the questionnaire package. Eight did not complete any survey questions, resulting in a final sample of 95 (pre-pandemic = 57; pandemic-born = 38).

#### 3.1.1. Child Development and Pregnancy Experience

**Figure 2:**
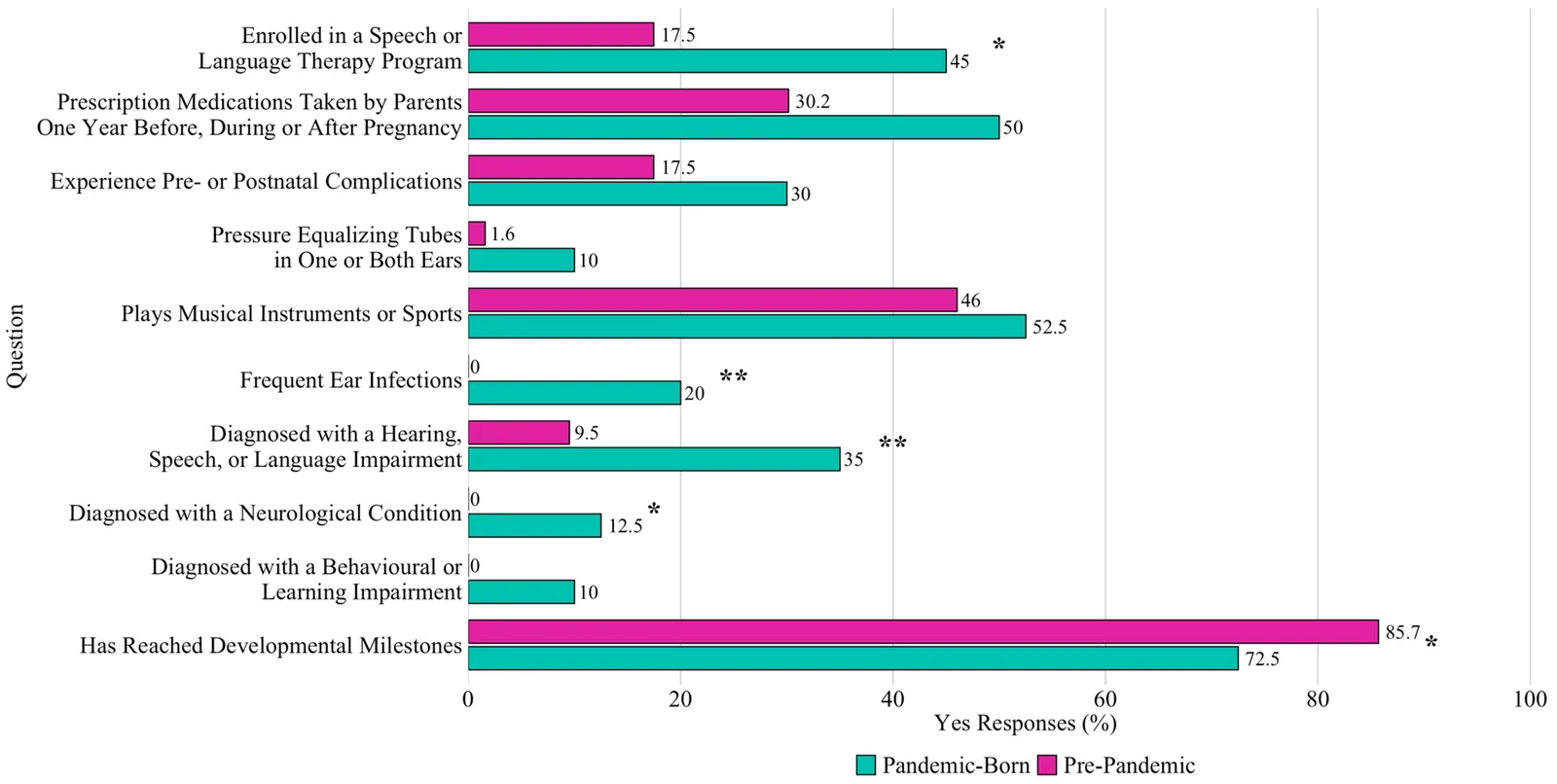
Child development and pregnancy experience survey responses by cohort. The figure shows the percentage of participants who responded ‘Yes’ out of all participants who selected either ‘Yes’ or ‘No’ for each item in their respective cohort. * *p* < 0.05, ** *p* < 0.01.

Caregivers reported on a range of pregnancy and development concerns. Figure 2 shows the percentage of responses that were “yes” for each item, by cohort. Chi-square tests were conducted to examine associations between cohort and item responses; results are presented in Table 2.

#### 3.1.2. Parents’ Assessment of Protective Factors

Descriptive statistics for the PAPF are shown in Table 3. Protective factor levels were comparable across cohorts, with no significant differences in any domain. Across both groups, 74% of caregivers reported high protective factor index strength levels, as reported by the PAPF group averages.

#### 3.1.3. Adverse Childhood Experiences Survey

Exposure to adverse experiences is closely associated with elevated perceived stress levels, which may influence both caregiver and child outcomes. Thus, we asked whether there was a difference between caregiver ACE and child ACE scores across the two cohorts. A Mann–Whitney U test showed a significant difference in ACE self-report scores between the pandemic (*n* = 33, *Mdn* = 2, *IQR* = 1–4) and the pre-pandemic group (*n* = 54, *Mdn* = 1, *IQR* = 0–2), *U* = 1137, *p* = 0.027, *r* = 0.237. Specifically, caregiver ACE scores in the pandemic-born cohort were significantly higher than the pre-pandemic cohort. In addition, a Mann–Whitney U test showed no significant difference in ACE child-report scores between the pandemic group (*n* = 34, *Mdn* = 0, *IQR* = 0–1) and the pre-pandemic group (*n* = 52, *Mdn* = 0, *IQR* = 0–1), *U* = 912.5, *p* = 0.768, *r* = 0.032.

Furthermore, we investigated whether there was a correlation between the caregiver’s ACE score and their child’s EF score. In this study sample, Spearman correlations showed no significant relationship between the caregiver’s ACE score and the child’s BRIEF-P GEC, *r*(83) = 0.08, *p* = 0.475, Shift scale, *r*(83) = 0.02, *p* = 0.078, and Flexibility Index, *r*(83) = 0.10, *p* = 0.357. Lastly, we wanted to assess if the caregiver’s ACE score correlated with the child’s ASQ-3 fine motor skills. Spearman’s rank correlation showed no significant correlation between the caregiver’s ACE score and the child’s ASQ-3 fine motor skills, *r*(84) = −0.038, *p* = 0.726.

#### 3.1.4. Screen Time

Given increasing concerns regarding screen time, we reasoned that the pandemic-born cohort may report a higher screen time average at the time of assessment in comparison to the pre-pandemic cohort. A Mann–Whitney U test revealed no significant difference between daily screen time exposure across the pre-pandemic (*n* = 54, *Mdn* = 75, *IQR* = 60–120) and pandemic (*n* = 38, *Mdn* = 60, *IQR* = 41.2–112), *U* = 817.5, *p* = 0.091 cohorts (pre-pandemic daily average of *M* = 100.37 min, pandemic *M* = 76.58 min).

In addition, we examined whether screen time was correlated with the BRIEF-P outcomes. Spearman’s correlation revealed no significant relationship between screen time reports and GEC scores, *r*(88) = 0.091, *p* = 0.392. A correlation analysis between screen time and the Shift scale also revealed no significant relationship, *r*(88) = −0.197, *p* = 0.062. Similarly, the correlation between screen time and the Flexibility index was non-significant, *r*(88) = −0.052, *p* = 0.628. Lastly, a correlation analysis between screen time and ASQ-3 fine motor scores yielded a weak but significant negative relationship, *r*(89) = −0.264, *p* = 0.011, suggesting that children with higher screen time may have poorer fine motor skills. Together, the results indicate that screen time was not significantly associated with EF in this study population.

### 3.2. Between-Group Differences in Executive Function

**Figure 3:**
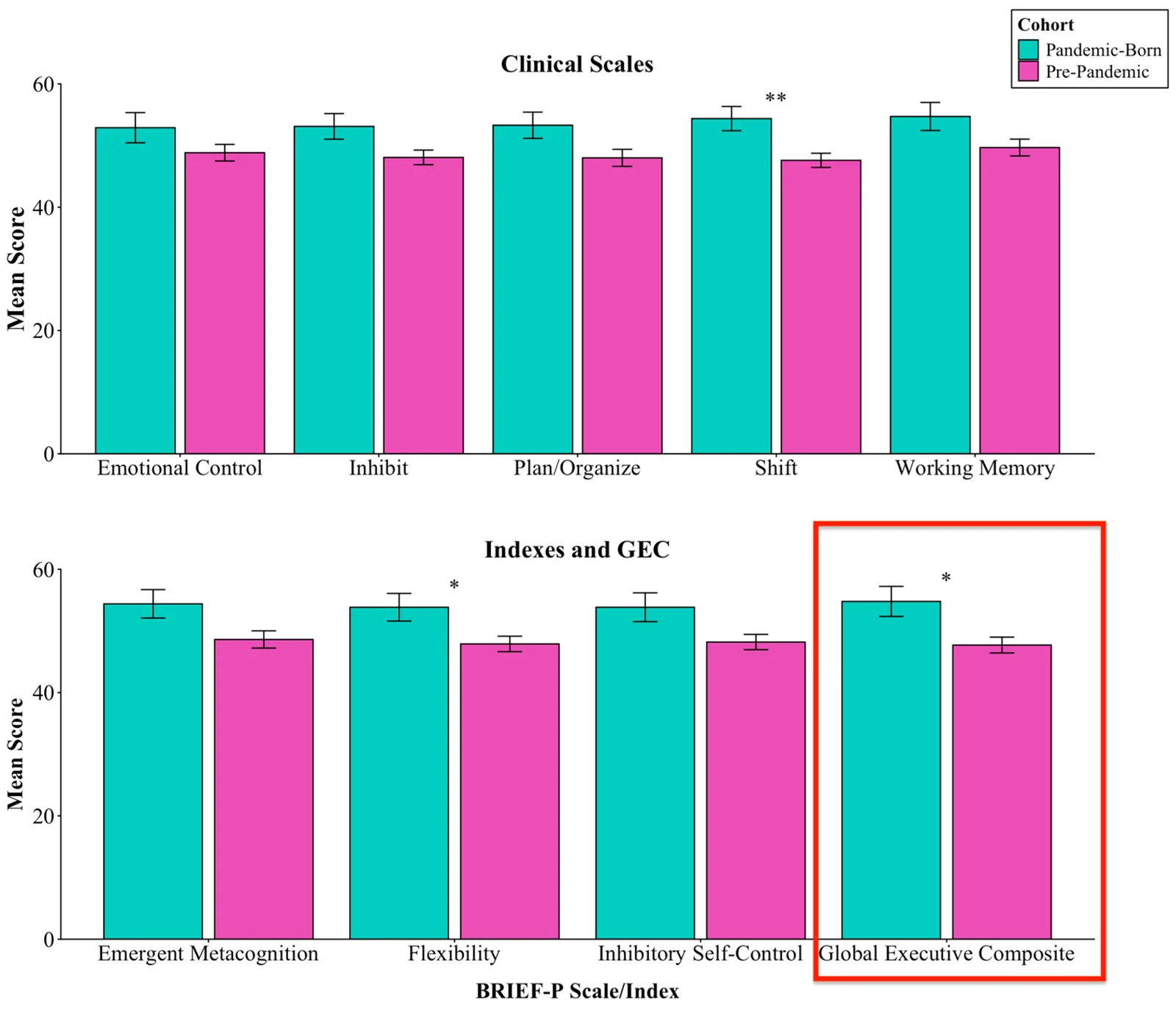
Behavior Rating Inventory of Executive Function—Preschool Version *T* scores across cohorts. Bar graph of BRIEF-P clinical scales (**top**) and indexes (**bottom**). High scores indicate low performance. Boxed area in this figure highlights the summary composite, which incorporates all five clinical scales of the BRIEF-P. * *p* < 0.05, ** *p* < 0.01.

We then compared EF skills between the pre-pandemic and pandemic-born cohorts. Mann–Whitney U tests were conducted to examine group differences on BRIEF-P scales and indexes, with lower *T* scores indicating better EF abilities. As shown in Figure 3, the pandemic-born cohort scored significantly higher across all scales and indexes. Significant differences were noted in the Shift scale, Flexibility index, and the GEC, suggesting lower EF capabilities in the pandemic-born relative to the pre-pandemic cohort (see Table 4 for descriptive and inferential statistics; see Table A1 for mean and standard deviation values).

### 3.3. Between-Group Differences in Developmental Screening Tools

**Figure 4:**
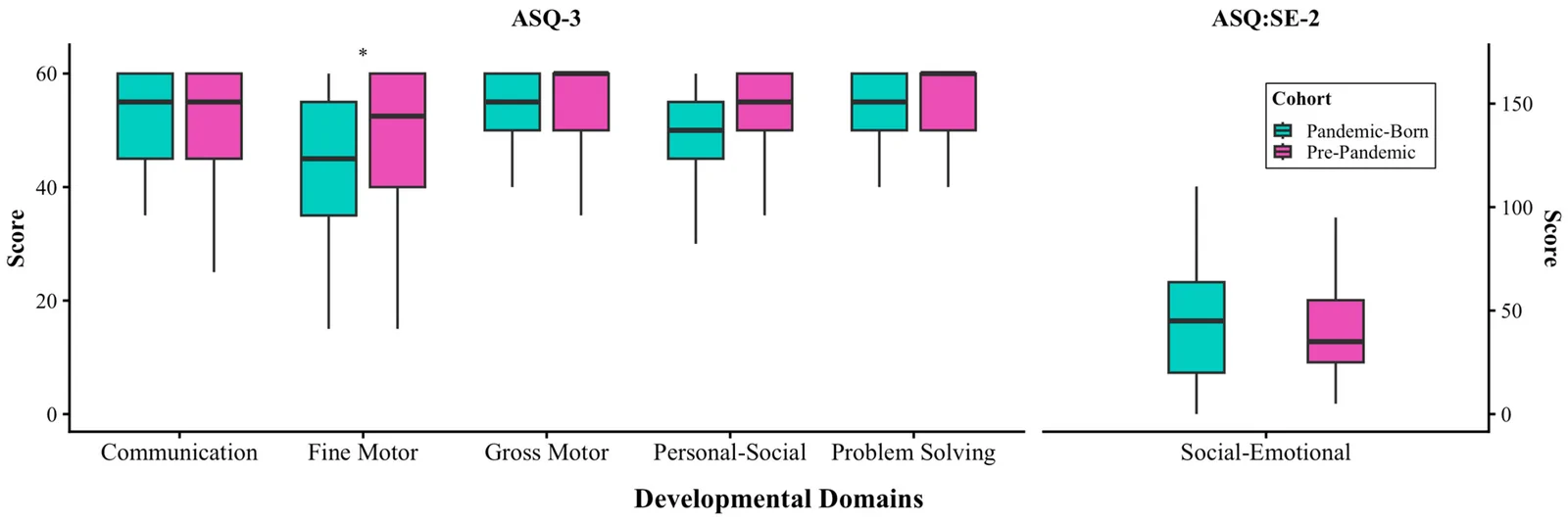
Boxplot distributions of total scores between groups on the ASQ-3 (**left**) and ASQ:SE-2 (**right**). Higher scores on the ASQ-3 domains indicate better performance, whereas lower scores on the ASQ:SE-2 social emotional domain indicate better performance. The bottom of the box represents the 25th percentile (Quartile 1), the top represents the 75th percentile (Quartile 3), and the line inside the box represents the median. Whiskers extend to the lowest and highest observations within 1.5 interquartile ranges. * *p* < 0.05.

Typical child development was assessed using the ASQ-3 and ASQ:SE-2, standardized measures of child development. Figure 4 displays boxplots showing the distribution of the two groups across both standardized screening tools. As shown in Table 5, a Mann–Whitney U test revealed a significant group difference in the ASQ-3 fine motor domain, with the pandemic-born scoring lower than the pre-pandemic cohort. Mann–Whitney U tests showed no significant group difference in the communication, gross motor, personal-social, or problem-solving domains. However, across all domains, the pandemic-born cohort exhibited lower performance relative to the pre-pandemic cohort. For the ASQ:SE-2, no statistically significant group differences were found in the social-emotional domain, as revealed by a Mann–Whitney U test. In this questionnaire, a lower score indicates better performance. As shown in Table 5, the pre-pandemic cohort displayed on average better performance scores than the pandemic-born cohort in the social-emotional domain. See Table A2 for mean and standard deviation values.

### 3.4. Between-Group Differences in Vocabulary Skills

**Figure 5:**
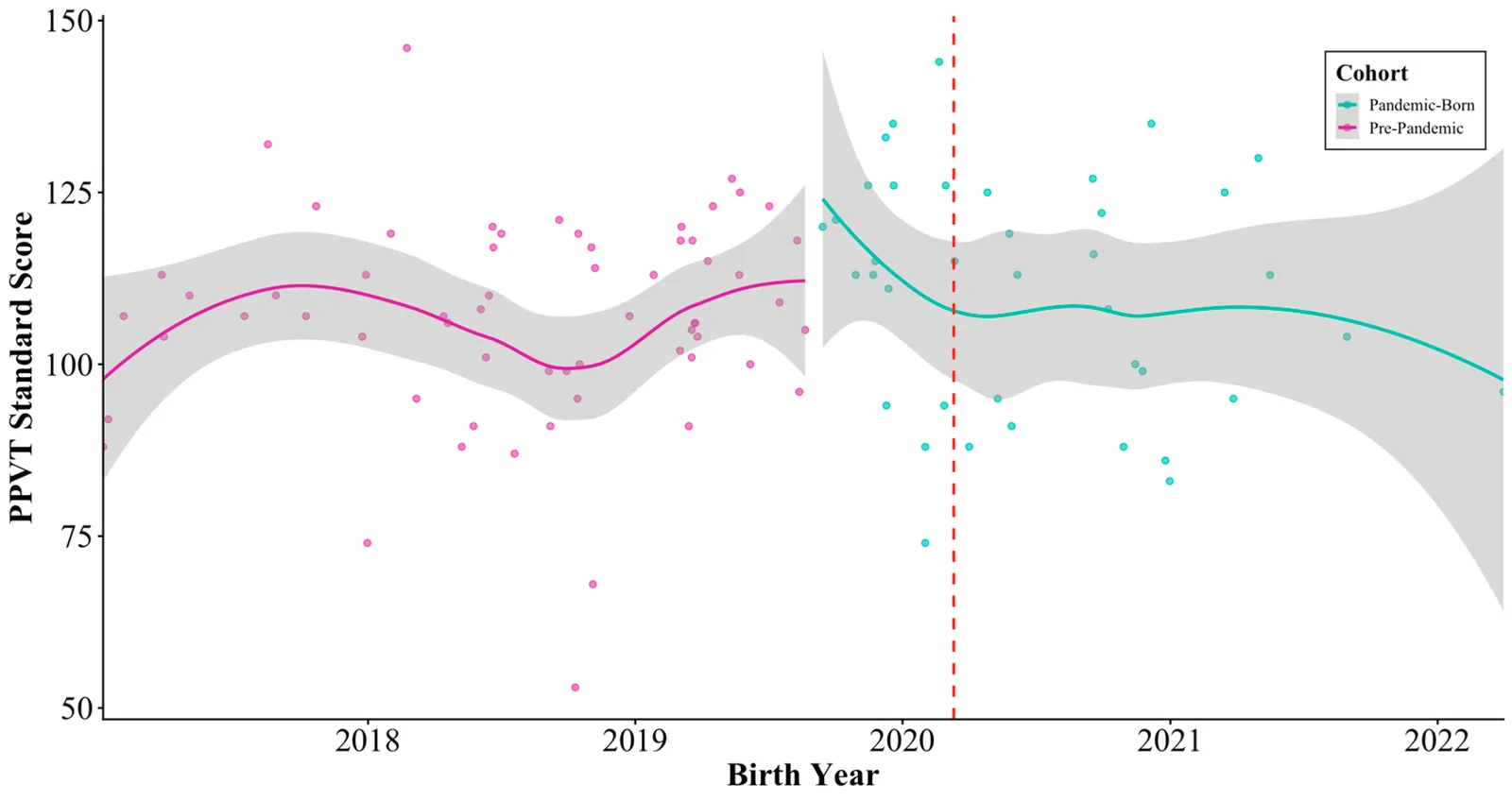
Distribution of PPVT-V standard scores across birth year relative to COVID-19 start. Points represent individual participants with a smoothed LOESS trend for each cohort with 95% confidence interval. The dashed line represents the date, 11 March 2020, at which COVID-19 was assessed as a pandemic.

Lastly, we were interested in whether vocabulary skills, as measured by the PPVT-V, differed between the pandemic-born and pre-pandemic cohorts. Data met the assumptions for a parametric test, so a Welch’s two-sample *t*-test was conducted. The results indicated that there was no significant difference in PPVT-V scores between the pandemic-born (*M* = 110.41, *SD* = 17.2) and pre-pandemic (*M* = 106.76, *SD* = 15.1) cohorts, *t*(72.99) = 1.09, *p* = 0.28, 95% CI [–3.03, 10.34]. As shown in Figure 5, there was a noticeable dip in PPVT-V scores for children born between 2018 and 2019, representing children that were 1–2 years of age at the start of the pandemic. An ANCOVA performed on PPVT scores across birth cohort while controlling for frequent ear infections yielded no significant effect, *F*(1, 88) = 0.53, *p* = 0.47. The covariate, frequent ear infections, was not significantly related to the PPVT scores, *F*(1, 88) = 0.00, *p* = 0.95. Therefore, frequent ear infections did not significantly influence PPVT scores.

## 4. Discussion

The rapid development in brain architecture during the early years is a period of heightened sensitivity to both opportunity and vulnerability. In the current study, we compared preschool-age developmental outcomes between children born before (2017–2019) and during (2019–2022) the COVID-19 pandemic. Both cohorts were assessed post-pandemic at 3–5 years of age. The pandemic-born cohort included children who were either in utero or up to five months old at its onset, whereas the pre-pandemic cohort were six months or older when the pandemic began.

To examine potential pandemic-related impacts during this age window, we first assessed child development and pregnancy experiences from the caregiver information sheet. Our findings indicate a decrease in developmental milestone achievement, as assessed by the Health Unit at vaccination appointments, among pandemic-born children. This cohort also showed increased rates of (1) hearing, speech, or language impairment, (2) enrollment in speech or language therapy programs, and (3) frequent ear infections. In contrast, we observed no group differences across cohorts in variables such as caregiver prescription medication use before, during or after pregnancy, child pre- or postnatal complications, pressure-equalizing tubes, screen time, and engagement in sports or musical instruments. Similarly, caregiver protective factors, as measured by the PAPF, were comparable across cohorts, indicating high parental resilience and support. Importantly, the absence of differences in these other factors strengthens our interpretation that the observed developmental differences may be related to pandemic factors rather than confounding variables. A surge in respiratory viral illnesses during this period likely contributed to an increase in related complications such as frequent ear infections. Consistent with these observations, Bacon et al. (2024) reported that children with a history of COVID-19 infection were at increased risk of recurrent acute otitis media or post-ventilation tube otorrhea compared to uninfected children. Importantly, both cohorts in our study were exposed to COVID-19, but the timing differed: pre-pandemic children were, on average, 18 months old at exposure, whereas pandemic-born children were in utero or up to five months old. These findings suggest that the age at first exposure may be a critical factor influencing recurrence. Early episodes of ear infections may have cascading effects, contributing to hearing, speech, or language impairment, greater enrollment in speech or language therapy programs and reduced developmental milestone achievement. This aligns with prior research indicating that recurrent otitis media in the first three years of life can be associated with delays in language, speech and cognition, although findings are mixed (Altamimi et al., 2023; Bennett & Haggard, 1999; Feldman et al., 2003; Paradise et al., 2000; Roberts et al., 2004; Winskel, 2006). While otitis media is common in early childhood, with up to 80% of children experiencing at least one episode by the age of three; those with onset before six months are more likely to experience recurrent episodes in the following two years than children whose first episode occurs after their first birthday (Teele et al., 1989). Collectively, these findings raise the possibility that pandemic-born children are more susceptible to recurrent ear infections and associated complications. Hence, the relationship between vocabulary skills and frequent ear infections among the pre- and pandemic born cohort was of considerable interest. In our study sample, we did confirm no relationship between birth cohort and PPVT scores after controlling for frequent ear infections. In addition, frequent ear infections were not predictive of vocabulary scores.

Other factors such as pandemic-related restrictions may contribute to differences in language development. Reduced social contact and the use of face masks may have limited recognition of familiar words and reduced the quality of conversational interactions (Lalonde et al., 2022). Pre-pandemic, 2-year-olds were exposed to 100–140 words per hour, compared with 20–70 words per hour during 2020–2021 (Sparks, 2022). In our sample, there was a slight group difference in PPVT scores, with the pandemic-born cohort, on average, scoring slightly higher than the pre-pandemic cohort. The differing exposure windows may have masked more robust group differences. Prior studies report mixed findings, with some indicating reduced vocabulary and language scores (Frota et al., 2022; Pejovic et al., 2024), while others observed positive effects mediated by increased parent-child interaction and reduced passive screen time (Kartushina et al., 2022). Although not a significant finding, we observed a dip in vocabulary scores among children born between 2018–2019, who were approximately 12 months old at the start of the pandemic. Interestingly, language milestone studies report that vocabulary acquisition begins around 12 months and shows a sharp acceleration around 16–18 months of age (Fenson et al., 1994; Nazzi & Bertoncini, 2003). There is a possibility that the dip in vocabulary skills reflects age-related differences in the developmental impact of the pandemic. Delays in vocabulary skills due to pandemic-related factors, such as limited social activities, may be most apparent during the rapid vocabulary spurt period. Overall, these findings suggest a cumulative effect of varied experiences on children’s language development during the COVID-19 pandemic.

We present clear evidence of group differences in EF scores between the pandemic-born and pre-pandemic cohorts. Using a standardized caregiver-report questionnaire, the BRIEF-P, we report poorer scores in the pandemic-born cohort on the Shift scale, which measures a child’s ability to move freely from one activity to another and solve problems flexibly; the Flexibility Index, which captures a broader capability to manage both behavioral and emotional responses to changing demands; and the GEC, an overall summary score derived from all five clinical scales. Across all scales and indices, we observed elevated *T*-scores in the pandemic-born cohort, indicative of poorer executive functioning. To our knowledge, no prior studies have measured or reported group differences in EF skills among preschool-aged children born during the pandemic. Existing research has primarily focused on behavioral and neurodevelopmental outcomes in infants under six months (Huang et al., 2021; Imboden et al., 2022; Shuffrey et al., 2022; Wu et al., 2021) and with short-term exposure to the pandemic, typically three or six months. Our findings extend the literature by highlighting that pandemic exposure may have lasting effects on EF skills assessed post-pandemic for those exposed to its onset in infancy.

Developmental milestones assessed via the ASQ-3 showed no significant group differences in communication, gross motor, problem solving and personal-social domains, nor in the ASQ:SE-2 social-emotional domain. However, pandemic-born children demonstrated poorer fine motor skills than their pre-pandemic peers. These mixed findings align with studies across different geographical locations. An Ireland-based study identified differences in communication skills at 12 (Byrne et al., 2023a) and 24 months (Byrne et al., 2023b) among children born during the pandemic with approximately three months of exposure. Shuffrey et al. (2022) reported group differences across gross motor, fine motor, and personal-social domains at six months in a New York sample, irrespective of maternal COVID-19 infection during pregnancy. In China, Wu et al. (2021) found no association between maternal COVID-19 infection during the last trimester and developmental delays in infants at three months, whereas Huang et al. (2021) identified differences in fine motor and communication domains using the Gessell Developmental Schedules among one-year old children, with no significant differences in six-month-old children using the ASQ-3. In Canada, an Ontario-based study administered the ASQ-3 at 24 and 54 months. At 24 months, the researchers reported better problem-solving and fine motor scores but poorer personal-social scores in the pandemic-exposed group (Finegold et al., 2023). At 54 months, pandemic-exposed children exhibited better visual memory, vocabulary, and overall cognitive performance compared to those assessed prior to the pandemic. These findings differ from our results, which indicate poorer fine motor skills in the pandemic-born cohort compared with the pre-pandemic cohort. It is important to clarify, however, that although the referenced study assessed children at 24 and 54 months during the pandemic period, the participants were not born during the pandemic (Finegold et al., 2023). Notably, this sample comprised families with higher socioeconomic status (SES) than the general Canadian population, implying that pandemic-related benefits may have been contingent on access to supportive, stimulating environments that foster optimal development. As explored by Hendry et al. (2022), home environments that provided enriching activities during COVID-19 were positively associated with children’s EF development. Similarly, an Illinois-based study (Imboden et al., 2022) found no overall differences among children assessed at 18, 24, and 36 months during the pandemic. However, slight declines in communication scores were noted among children assessed at 6 and 12 months, age groups that spent most of their lives entirely during the pandemic. Taken together, developmental differences appear most pronounced in cohorts with in utero exposure or those born during the pandemic, irrespective of geographical location or maternal COVID-19 infection. In the current study, group differences were observed in the ASQ-3 fine motor domain and the BRIEF-P measure of EF skills. The findings indicate that early exposure (beginning in utero) may be linked to less favorable developmental outcomes.

Pandemic-induced reductions in social and sensory stimulation, combined with increased caregiver stress, may have contributed to these effects. Childhood learning is fundamentally grounded in exploratory play, which supports the development of motor, cognitive, and social skills (Fenson & Schell, 1985). By approximately five months of age, infants enhance their range of sensorimotor experiences through retrieving objects within reach (Fenson & Schell, 1985). Consistent with this, children’s exploratory engagement with novel multi-function toys has been associated with higher cognitive outcomes, including IQ (Muentener et al., 2018). Furthermore, intentional adult-directed play has been shown to foster executive function development in the early years (Coelho et al., 2020). Within the context of the COVID-19 pandemic, unprecedented lockdown measures in Canada substantially altered children’s daily environments. Restrictions on social interaction included limited access to playgrounds, libraries, and other enrichment settings. However, the pandemic did not affect all families equally (Fung et al., 2023). Social and home environments, family demographics, parental resilience and perceived stress are shown to be influential factors in mitigating negative pandemic-related effects (Liu et al., 2022). For instance, families with a higher number of children and/or living in rural areas compared to densely populated urban areas were more likely to spend time engaging with family and playing outside (Liu et al., 2022). Interestingly, in our study sample, we observed a significant difference in caregiver ACE scores, with more adverse childhood experiences reported among caregivers of the pandemic-born cohort. No correlation was observed between the caregiver’s ACE score and the child’s EF outcome. Overall, the ACE scores in both the pandemic and pre-pandemic cohort were low, suggesting no negative transgenerational effects. The questionnaire has previously demonstrated strong evidence linking a score of six or more adverse childhood experiences to negative health outcomes in adulthood (Felitti et al., 1998). Past studies have also suggested a positive correlation between maternal ACE scores and perceived stress and mental health challenges during pregnancy (Fields et al., 2023). While the pandemic widely affected psychological well-being during pregnancy (Nazzari et al., 2024), vulnerable populations such as those with high ACE scores, may have been disproportionately impacted. These findings underscore the importance of longitudinal monitoring and support for vulnerable populations experiencing environmental stress. Furthermore, future studies should assess the impact of caregiver ACE scores as a potential factor underlying the degree of pandemic-related stress.

Several limitations should be considered when interpreting these findings. First, this study employed a retrospective design, which carries inherent constraints such as controlling all confounding variables. For instance, detailed information on the extent to which families adhered to social distancing or home confinement measures was unavailable. Data collection occurred after the pandemic; meaning that caregiver-reported items such as screen time, family composition, and protective factors reflect post-pandemic perspectives rather than contemporaneous experiences. EF and neurodevelopmental outcomes were assessed using caregiver-report measures. Although these instruments include validity checks to account for response inconsistency and negativity, caregiver responses may still be influenced by their own interpretations of statements. The pandemic-born cohort included children born between 2019 and 2022, representing variation in timing and duration of exposure. Some children experienced the pandemic primarily in utero, while others encountered it during infancy. All children were assessed during the preschool period and had varying windows of exposure to the COVID-19 pandemic. Children in the pre-pandemic born group were exposed to pandemic-related restrictions during later infancy, whereas children in the pandemic-born group were exposed during early infancy (i.e., before 5 months of age). Thus, neither group is entirely unexposed; rather, the groups differ in the timing of their exposure. This distinction should be considered when interpreting the results. Due to the modest sample size, subgroup analyses by birth year could not be conducted. Future research should examine these groups separately, ideally using larger, longitudinal samples and age-specific exposure bins to clarify sensitive developmental periods. Overall, small samples also increase the likelihood of underpowered or false-negative results (Maxwell, 2004). Additionally, both the pre- and pandemic born cohorts were recruited from a predominantly Caucasian, rural region. Replication in larger and more diverse urban settings where restrictions and community transmission occurred at different intensities would help determine the generalizability of these results. Finally, the present study did not address which specific factors within the complex set of COVID-19 related changes contributed to developmental differences.

## 5. Conclusions

This study offers a unique window into the associations between early-life during COVID-19 and children’s neurodevelopment at the preschool age. Taken together, the results suggest that, in our sample, the pandemic-born cohort exhibited a higher frequency of ear infections, greater enrollment in speech and language therapy programs, delays in reaching developmental milestones and poorer scores in EF and fine motor skills. In this study, we observed a significant difference in caregiver ACE scores, with higher scores in the pandemic-born cohort suggesting increased levels of perceived stress during the pandemic as a contributing factor. Importantly, these associations are likely to be shaped by broader contextual factors, including family demographics and access to resources. Variability in parental engagement, availability of early intervention services, and childcare support during the pandemic may have influenced both children’s developmental trajectories and families’ capacity to mitigate pandemic-related stressors. As such, the observed effects may not be uniform across populations and may be exacerbated in contexts with greater structural barriers and vulnerable populations. Further research is warranted to elucidate the factors underlying poorer developmental outcomes among pandemic-born children. Overall, while these findings raise potential concerns about early developmental trajectories of children born during the COVID-19 pandemic, long-term follow-up studies are necessary to determine whether these effects persist into formal schooling.

## Figures and Tables

**Table 1:** Sociodemographic characteristics of the study population by cohort.

Sociodemographic Characteristics	Pre-Pandemic *n* = 63	Pandemic-Born *n* = 40
*M* (*SD*) Age at time of testing, y	4.45 (0.75)	4.23 (0.60)
Median (*IQR*)	4.40 [3.70, 4.99]	4.28 [4.00, 4.58]
*M* (*SD*) Age relative to pandemic start ^a^, y	−1.59 (0.72)	0.31 (0.60)
Median (*IQR*)	−1.45 [−2.03, −0.98]	0.18 [−0.23, 0.71]
*M* (*SD*) Age relative to pandemic start ^a^, d	−580.95 (262.23)	114.90 (218.03)
Median (*IQR*)	−530 [−742, −359]	67 [−85, 260]
Sex, *n* (*%*)		
Male	30 (47.6)	20 (50.0)
Female	33 (52.4)	20 (50.0)
Race, *n* (*%*)		
Caucasian, White, or European American	42 (68.9)	30 (76.9)
African American or Black	4 (6.6)	0
Asian	7 (11.5)	2 (5.1)
Hispanic or Latino	3 (4.9)	1 (2.6)
Biracial or Multiracial	4 (6.6)	5 (12.8)
Other	1 (1.6)	1 (2.6)
Caregiver Education, *n* (*%*)		
No Formal Education	1 (1.8)	1 (2.7)
High School	7 (12.5)	4 (10.8)
Trade/Technical School	4 (7.1)	3 (8.1)
1 year college certificate	1 (1.8)	0
2-year college associate’s degree	10 (17.9)	6 (16.2)
4-year bachelor’s degree	25 (44.6)	13 (35.1)
Post-Graduate Degree	8 (14.3)	10 (27.0)
Household Composition, *n* (*%*)		
Nuclear Family	53 (92.9)	33 (89.2)
With Siblings	45 (78.9)	27 (73.0)
Without Siblings	8 (14.0)	6 (16.2)
Single Parent	2 (3.5)	2 (5.4)
With Siblings	0 (0)	1 (2.7)
Without Siblings	2 (3.5)	1 (2.7)
Non-Parental Care	2 (3.5)	2 (5.4)

Note: *N* = 103. Interquartile range (*IQR*; 25th—75th percentile). ^a^ Values reflect participants’ birth dates relative to the date COVID-19 was declared a pandemic (11 March 2020). Positive values indicate days after this date, and negative values indicate days before.

**Table 2:** Chi-square analysis of child development and pregnancy experience.

Question	X^2^	df	*p*
Has your child ever participated in speech or language therapy programs?	7.07	1	0.008
2. Was either parent taking any prescription medications one year prior to conception, during pregnancy, or one year after childbirth?	2.76	1	0.097
3. Did your child experience any pre-or post-natal complications?	1.26	1	0.261
4. Does your child have pressure equalizing tubes in one or both ears in order to prevent ear infection?	1.86	1	0.173
5. Does your child play any sports or musical instruments?	0.03	1	0.87
6. Does your child have frequent ear infections?	10.13	1	0.001
7. Has your child ever been diagnosed with a hearing, speech, or language impairment?	7.91	1	0.005
8. Has your child ever been diagnosed with any neurological condition with sensory/motor consequences, such as attention deficit hyperactive disorder (ADHD), cerebral palsy, multiple sclerosis, Tourette’s syndrome, epilepsy, developmental coordination disorder (DCD), or other?	5.28	1	0.022
9. Has your child ever been diagnosed with a behavioral or learning impairment?	3.76	1	0.052
10. Did your child reach all of his/her developmental milestones on time (those that are assessed by the Health Unit at vaccination appointments)?	5.44	1	0.020

Note: significant values (*p* < 0.05) compare item responses across birth cohorts. The direction of differences can be further confirmed in Figure 2.

**Table 3:** Comparison of parents’ assessment of protective factors across cohorts.

	Pre-Pandemic *n* = 54	Pandemic-Born *n* = 38
**PAPF Total Scores**	*M* (*SD*)	*M* (*SD*)
Parental Resilience	30.5 (4.2)	31.1 (4.5)
Average Score	3.4 (0.47)	3.5 (0.50)
Social Connections	29.0 (5.2)	29.9 (6.4)
Average Score	3.2 (0.58)	3.3 (0.71)
Concrete Support in Times of Need	28.8 (5.2)	28.5 (5.6)
Average Score	3.2 (0.58)	3.2 (0.62)
Social and Emotional Competence of Children	27.9 (3.8)	29.7 (4.5)
Average Score	3.1 (0.42)	3.3 (0.50)
Protective Factors Index Total	116.1 (13.6)	119.2 (15.7)
Average Score	3.2 (0.38)	3.3 (0.44)
**Protective Factors Index Strength Level, ***n*** (%)**		
Low	1 (1.9)	0
Moderate	12 (22.2)	9 (23.7)
High	40 (74.1)	28 (73.7)
Maximum	1 (1.9)	1 (2.6)

**Table 4:** Inferential statistics for BRIEF-P questionnaire.

BRIEF-P Score	Total *Mdn* (*IQR*)	Pre-Pandemic *Mdn* (*IQR*)	Pandemic-Born *Mdn* (*IQR*)	*U*	*p*
Scale/Index					
Inhibit	48 (42–57)	48 (41–54)	50 (43–58.75)	1257.5	0.097
Shift	49 (42–55)	47 (40.5–52.5)	54 (43.5–61.25)	1399	0.005
Emotional Control (EC)	49 (41–56)	48 (41–55)	52 (41–56)	1186	0.271
Working Memory (WM)	48 (44–58)	48 (42–56)	50 (46–61.5)	1257.5	0.097
Plan/Organize (PO)	49 (41–54)	48 (41–52.5)	51 (44.5–60)	1290.5	0.055
ISCI (Inhibit + EC)	49 (42–55)	48 (41.5–52)	54 (42–57.75)	1263.5	0.088
FI (Shift + EC)	47 (41–57)	47 (39.5–55.5)	50.5 (44–59.25)	1298	0.048
EMI (WM + PO)	49 (42–58)	48 (41–52.5)	50.5 (43.75–62)	1293	0.053
GEC (Inhibit + Shift + EC + WM + PO)	48 (42–56)	46 (41–54)	50 (43.5–60)	1328	0.027

Note: Pre-pandemic (*n* = 55), pandemic-born (*n* = 38) and total (*N* = 93). Interquartile range (*IQR*; 25th–75th percentile).

**Table 5:** Descriptive and inferential statistics for standardized developmental screening tools.

Developmental Domains	Total *Mdn* (*IQR*)	Pre-Pandemic *Mdn* (*IQR*)	Pandemic-Born *Mdn* (*IQR*)	*U*	*p*
**ASQ-3**					
Communication	55 (45–60)	55 (45–60)	55 (45–60)	962	0.463
Fine Motor	50 (40–55)	52.5 (40–60)	45 (35–55)	759.5	0.018
Gross Motor	57.5 (50–60)	60 (50–60)	55 (50–60)	933.5	0.282
Personal Social	50 (45–60)	55 (50–60)	50 (45–55)	852.5	0.095
Problem Solving	55 (50–60)	60 (50–60)	55 (50–60)	999	0.595
**ASQ:SE-2**					
Social Emotional	40 (25–57.5)	35 (25–55)	45 (20–65)	1107	0.384

Note: ASQ-3 scores are reported for the pre-pandemic (*n* = 56) and pandemic (*n* = 38) groups, with a total sample of *N* = 94. ASQ:SE-2 scores are reported for the pre-pandemic (*n* = 54) and pandemic (*n* = 37) groups, with a total sample of *N* = 91. Interquartile range (*IQR*) reflects the 25th–75th percentiles.

## Data Availability

The datasets presented in this article are not readily available because the data are part of an ongoing study and due to the privacy of the research participants. Requests to access the datasets should be directed to the corresponding author.

## Supplementary Materials

The following supporting information can be downloaded at: https://www.mdpi.com/2076-328X/16/2/309/s1. S1: Participant Information Sheet.

## Appendix A

**Table A1:** Descriptive statistics for BRIEF-P questionnaire.

BRIEF-P Score	Pre-Pandemic *M* (*SD*)	Pandemic-Born *M* (*SD*)	Total *M* (*SD*)
Scale/Index			
Inhibit	48.1 (8.8)	53.1 (12.8)	50.1 (10.8)
Shift	47.60 (8.5)	54.4 (12.1)	50.4 (10.6)
Emotional Control (EC)	48.8 (10.0)	52.9 (15.1)	50.5 (12.4)
Working Memory (WM)	49.7 (10.1)	54.7 (14.0)	51.7 (12.1)
Plan/Organize (PO)	48.0 (10.2)	53.3 (13.1)	50.2 (11.7)
ISCI (Inhibit + EC)	48.2 (9.2)	53.8 (14.4)	50.5 (11.9)
FI (Shift + EC)	47.9 (9.3)	53.8 (13.8)	50.3 (11.7)
EMI (WM + PO)	48.6 (10.4)	54.4 (14.2)	51.0 (12.4)
GEC (Inhibit + Shift + EC + WM + PO)	47.7 (9.5)	54.8 (15.0)	50.6 (12.5)

**Table A2:** Descriptive statistics for ASQ-3 and ASQ:SE-2 questionnaires.

Developmental Domains	Pre-Pandemic *M* (*SD*)	Pandemic-Born *M* (*SD*)	Total *M* (*SD*)
**ASQ-3**			
Communication	51.1 (9.3)	49.6 (13.6)	50.5 (11.2)
Fine Motor	48.2 (12.7)	41.6 (15.6)	45.5 (14.2)
Gross Motor	54.5 (8.0)	51.8 (12.0)	53.4 (9.9)
Personal Social	52.1 (8.5)	47.6 (13.1)	50.3 (10.8)
Problem Solving	54.6 (7.7)	53.8 (8.26)	54.3 (7.9)
**ASQ:SE-2**			
Social Emotional	40.6 (21.1)	49.7 (35.7)	44.3 (28.1)
