# Supplementary material for: Pandemic Babies: Developmental Outcomes in Preschool-Aged Children Born During the COVID-19 Era
Source: Behav Sci (Basel). 2026 Feb 23;16(2):309. doi: 10.3390/bs16020309 (PMC12937897; doi:10.3390/bs16020309)
Supplement: Supplementary file 1 [file behavsci-16-00309-s001.zip › behavsci-4045308-supplementary.pdf]

Robbin Gibb, PhD
Associate Professor
Department of Neuroscience
The University of Lethbridge
4401 University Dr.
Lethbridge Alberta
T1K 3M

The Brain in Action lab
Department of Kinesiology
University of Lethbridge
4401 University Dr. W
Lethbridge, Alberta
T1K 4E1

Participant Information Sheet

Please fill out this brief questionnaire on behalf of your child and hand it in along with the consent form.

SECTION A - General

Child name: ____________________________ Gender:___ Date of Birth:______________ Age: ____ Grade: ___

Your name: _______________________ Telephone: __________________ Postal Code: ______________

Email: __________________________________ What is your child's ethnicity? _____________________

Where was your child born? _____________________ How long has your child lived in Lethbridge? ____________

Are any of your child's family members native speakers of languages other than English? Y ___ N ___

If yes, who are they and what language do they speak? _______________________________________

Please describe your child's family composition, i.e. who lives in the home with the child? For example: "*mother, father, grandmother, 2 brothers*." For siblings, please list ages.

____

____

Did your child experience any pre- or post-natal complications (e.g. premature birth)? Y ____ N ____
If yes, please explain.

____

____

If your child was born pre-maturely (i.e., less than or equal to 37 weeks) please indicate the weeks at which they were born (e.g. 28 weeks) _________

If your child was born at low birth weight (i.e., less than or equal to 2500g) please indicate their weight in grams (e.g. 2500g) ___________

Was either parent taking any prescription medications one year prior to conception, during pregnancy, or one year after childbirth? Y ____ N ____

If yes, please list the medications, the length of use, and at what point (e.g. prior, during, post) the medication was used.

____

____

Did your child reach all of his/her developmental milestones on time (those that are assessed by the Health Unit at vaccination appointments)? Y ____ N ____

If no, please specify which milestone(s) was not reached on time, and when it was achieved.

____

____

Does your child have frequent ear infections? Y ___   N ___

Does your child have PE tubes in one or both ears in order to prevent ear infection?   Y ___   N ___

SECTION B – Motor

Has your child ever been diagnosed with any neurological condition with sensory/motor consequences, such as attention deficit hyperactive disorder (ADHD) cerebral palsy, multiple sclerosis, Tourette's syndrome, epilepsy, developmental coordination disorder (DCD), or other? Y ____ N____
If yes, please describe the diagnosis, age of your child at diagnosis, and any treatment:

____

____

Does your child play any sports or musical instruments? Y_____ N_____

If yes, please explain. For each sport/instrument, please include the age of your child when he/she began to play and frequency of practice.

____

____

____

SECTION C – Executive Function

Has your child ever been diagnosed with a behavioral or learning impairment? Y ___ N___
If yes, please describe the diagnosis, age of your child at diagnosis, and any treatment(s):

____

____

SECTION D - Language

Has your child ever been diagnosed with a hearing, speech, or language impairment? Y ___ N ___

If yes, please describe ____

Has your child ever participated in speech or language therapy programs? Y ___ N ___

If yes, please describe ____

If yes, what age did they begin therapy? _________ What age did they complete therapy? _________

Would you like to be put in our mailing list for future studies?   Y ____ N ____

Thank you for participating in this study!
- Drs. Claudia Gonzalez, Robbin Gibb, Sally Sade
